# Olanzapine long-acting injection: a review of first experiences of post-injection delirium/sedation syndrome in routine clinical practice

**DOI:** 10.1186/s12888-015-0450-9

**Published:** 2015-04-02

**Authors:** Chris J Bushe, Deborah Falk, Ernie Anand, Marta Casillas, Elena Perrin, Rashna Chhabra-Khanna, Holland C Detke

**Affiliations:** Eli Lilly, Lilly House, Priestly Road, Basingstoke, Hampshire RG24 9NL UK; Eli Lilly, Lilly Corporate Center, Indianapolis, IN 46285 USA; Eli Lilly, Erlwood, Windlesham, Surrey GU20 6PH UK; Eli Lilly, Avda. de la Industria 30, 28108 Alcobendas, Madrid Spain; Eli Lilly, 13, Rue Pages, 92158 Suresnes Cedex, Paris France

**Keywords:** Depot antipsychotic, Olanzapine, Olanzapine long-acting injection, Olanzapine pamoate, Post-injection delirium/sedation syndrome, Schizophrenia

## Abstract

**Background:**

Olanzapine long-acting injection (LAI) for the treatment of schizophrenia was associated with a cluster of symptoms termed post-injection delirium/sedation syndrome (PDSS) in a small percentage (~2%) of patients during clinical trials. The objective of this analysis was to evaluate the rate and clinical characteristics of PDSS since olanzapine LAI entered commercial use.

**Methods:**

Cases of PDSS were identified from all reported adverse events during worldwide commercial use of olanzapine LAI through to 1 March 2014. Data sources included two ongoing post-marketing safety studies as well as spontaneously reported adverse events from routine clinical practice over a 5-year period (1 March 2009 to 1 March 2014).

**Results:**

A total of 338 PDSS events were identified. Of these, 91% occurred within 1 hour of injection, and 52% of these occurred within 15 minutes. None of the PDSS events in this analysis were fatal, and most resolved within 72 hours. The most common symptoms (occurring in >30% of cases) were sedation (61%), confusion (56%), dysarthria (54%), somnolence (46%), dizziness (45%) and disorientation (35%). Overall, PDSS occurred with approximately 0.07% of injections and in 0.46–1.03% of patients (reporting and incidence rates from spontaneous reports and post-marketing safety studies, respectively).

**Conclusions:**

The PDSS events reported during routine clinical use of olanzapine LAI are generally similar in incidence and presentation to those reported in clinical trials. Caution should be applied when interpreting spontaneously reported rates of adverse events, however, due to potential under-reporting. Implemented risk-minimisation activities may contribute substantially to the identification and appropriate management of patients with PDSS in clinical practice.

**Electronic supplementary material:**

The online version of this article (doi:10.1186/s12888-015-0450-9) contains supplementary material, which is available to authorized users.

## Background

Compliance with oral medication is often poor in patients suffering from illnesses such as schizophrenia, leading to relapse and subsequent hospitalisation [1,2]. Over the past decade, a number of atypical antipsychotics have been developed as long-acting injectable depot formulations, with the intention of enhancing patient compliance and improving health outcomes [1,2]. Olanzapine long-acting injection (LAI) consists of a pamoate salt of olanzapine that is administered by deep intramuscular injection every 2–4 weeks. It has been available in 31countries for over 5 years for the treatment of schizophrenia in adults.

Clinical trials have shown that olanzapine LAI has comparable efficacy and safety to oral olanzapine with the exception of adverse events related to the route of administration [3-5]. In these trials, olanzapine LAI was associated with a cluster of symptoms related to post-injection delirium and/or excessive sedation in a small number of patients (~2%) [5-8]. These symptoms, collectively referred to as post-injection syndrome or post-injection delirium/sedation syndrome (PDSS), have been fully defined (see list below) and appear to be consistent with symptoms associated with an oral olanzapine overdose [5,6].

For a clinical diagnosis of PDSS, the following criteria must be met [6]:

1. One or both of the conditions listed in (a) and (b):
  - A minimum of 1 sign or symptom from at least 3 of the following symptom clusters consistent with olanzapine^a^ overdose with one or more of at least moderate severity:
    Sedation/somnolence
    Delirium/confusion/disorientation/other cognitive impairment
    Dysarthria/other speech impairment
    Ataxia/other motor impairment
    Extrapyramidal symptoms
    Agitation/irritability/anxiety/restlessness
    Dizziness/weakness/general malaise
    Seizure
  - Any 1 of the following signs and symptoms:
    Unarousable
    Unconscious
    Stuporous
    Comatose
2. Condition develops within 24 hours of an olanzapine LAI.
3. Condition cannot be explained by a significant dose increase of olanzapine LAI, initiation or addition of oral olanzapine or other sedating medication, or new exposure to olanzapine LAI.
4. Underlying medical conditions have been ruled out, including concomitant substance use or abuse.

PDSS is thought to arise as a result of accidental entry of olanzapine into the bloodstream due to blood vessel injury during the injection process [9], a documented risk for intramuscular injections [10-13]. Special precautions for olanzapine LAI administration are therefore recommended [6], and two post-marketing safety studies are underway to further evaluate the frequency, signs, symptoms, clinical characteristics and outcomes of PDSS in routine clinical practice.

We present an interim 5-year cohort analysis of reported cases of PDSS from ongoing post-marketing safety studies of olanzapine LAI, together with all cases spontaneously reported from the time of first commercial availability of olanzapine LAI through to 1 March 2014. Our objective was to investigate the occurrence rate and clinical characteristics of PDSS in routine clinical practice and to compare these with the rates and characteristics reported in formal clinical trials.

## Methods

### Overview

Cases of PDSS were identified from two post-marketing safety studies and from spontaneous reports of adverse events from the first introduction of olanzapine LAI to the market, 1 March 2009, through to 1 March 2014 (a period of 5 years), as outlined below. All reports of adverse events, including all suspected cases of PDSS, were reviewed by an adjudication committee for classification as possible cases of the syndrome using published criteria (Table 1) [6].

**Table 1:** **Number of cases and rate of PDSS in routine clinical practice**

**Parameter**	**Post-marketing safety studies**		**Post-marketing safety studies combined**	**Spontaneous reports**	**Overall** ^**a**^
	**Global observational study (B034)**	**US Patient Care Program Registry (B041)**			
Total number of injections	38,721	49,991	88,712	411,209^b^	499,921^c^
Total number of patients	2131	4001	6132	58,868^d^	65,000^d^
Number of PDSS events	19 (in 18 patients)	47 (in 45 patients)	66 (in 63 patients)	272 (in 270 patients)	338
Rate of PDSS (% of injections)	0.05	0.09	0.07	0.07^e^	N/A^f^
Rate of PDSS (% of patients)	0.84	1.1	1.03	0.46^g^	N/A^f^

^a^Post-marketing safety studies and spontaneous reports combined.

^b^Estimated by excluding the number of vials used in the post-marketing safety studies, and assuming that only 75% of all vials sold are used.

^c^Includes estimated number of injections administered outside of the post-marketing safety studies.

^d^Estimated number of patients receiving an injection outside of the post-marketing safety studies.

^e^Based on the estimated number of injections administered outside of the post-marketing safety studies.

^f^ Not applicable; actual rates from post-marketing safety studies and estimated rates from spontaneous reports cannot be combined.

^g^Based on the estimated number of patients receiving an injection outside of the post-marketing safety studies.

PDSS = post-injection delirium/sedation syndrome.

### Post-marketing safety studies

#### Global observational study

Study F1D-MC-B034 (B034) is an ongoing multinational surveillance study designed to assess the incidence of PDSS in patients with schizophrenia treated with olanzapine LAI in real-world clinical practice. The study also aims to characterise the clinical presentation and outcomes of PDSS, and to provide insight into the possible predictors and risk factors, if any exist. All patients are ≥18 years of age with a diagnosis of schizophrenia and are currently receiving olanzapine LAI in accordance with their physician’s standard of care. Patient treatment, dosing and adverse event management are at the discretion of the investigator.

The study was approved in all countries either at the site, regional, or national level, depending on the country and local regulations. Patient consent followed country regulations. The study protocol was approved by the Ethical Review Board at each study centre Additional file 1. The study is being conducted in full accordance with the Declaration of Helsinki, Good Clinical Practice and applicable laws or regulations.

#### US patient care program registry

Study F1D-MC-B041 (B041) is an ongoing Patient Care Program Registry in the US that collects data from each olanzapine LAI administration in the country, such as dose, date, time of administration and signs or symptoms of PDSS. All prescribers, patients, healthcare facilities and pharmacy service providers, who administer, or receive olanzapine LAI in the US, are enrolled in the programme and receive the necessary education and training to recognise PDSS. Multiple methods are used to enrol and track participants, and follow-up is performed if further information is required to characterise a PDSS event.

### Spontaneously reported cases of PDSS

Spontaneously reported cases of PDSS were captured from Eli Lilly’s worldwide pharmacovigilance system. This system collects, monitors, evaluates and communicates information about adverse events spontaneously reported to Eli Lilly. All reports of adverse events in patients treated with olanzapine LAI were assessed to determine whether a PDSS event could have occurred and whether the criteria for PDSS had been met using the published criteria [6].

### Data extraction and analysis

For the post-marketing safety studies, data extracted included the total number of actively enrolled patients, the total number of injections administered, and the total number of PDSS events. The rate of PDSS was calculated as a percentage of the number of injections administered and as a percentage of the number of patients receiving at least one injection.

For spontaneously reported data, the rate of PDSS was calculated as a percentage of the estimated number of injections administered and as a percentage of the estimated number of patients receiving at least one injection. The number of injections was estimated based on 75% of the total number of olanzapine LAI vials sold minus the number of vials used in the post-marketing safety studies. The number of patients receiving an injection was extrapolated from olanzapine LAI usage patterns in the US Patient Care Program Registry.

Additional data on PDSS extracted from the post-marketing safety studies and spontaneous reports included the following: number of injections received before the occurrence of PDSS; the time to initial onset of PDSS; hospitalisation status; treatment received for PDSS; recovery status; and symptoms. Descriptive statistics were used to characterise the clinical presentation of PDSS, including outcomes.

## Results

### Overview

A total of 338 PDSS events were identified in 333 patients treated with olanzapine LAI between 1 March 2009 and 1 March 2014. Of the 338 events, 66 were sourced from the two post-marketing safety studies, and 272 were spontaneous reports. The estimated number of injections administered worldwide during this time was 499,921 and the estimated number of patients receiving an injection worldwide was 65,000 (Table 1).

### Post-marketing safety studies

A total of 88,712 injections of olanzapine LAI were administered to 6132 patients in the two post-marketing safety studies. Sixty-six PDSS events were reported in 63 patients, indicating that PDSS occurred with approximately 0.07% of injections, and in 1.03% of patients in these post-marketing studies (Table 1).

### Spontaneously reported cases of PDSS

Outside of clinical trials and the post-marketing safety studies, it was estimated that 411,209 injections were administered to 58,868 patients. A total of 272 PDSS events were reported in 270 patients, indicating that PDSS occurred with approximately 0.07% of injections and in 0.46% of patients according to spontaneous reports (Table 1).

### Number of injections before occurrence of PDSS

For both the post-marketing safety studies and spontaneous data, PDSS events were reported most commonly after the first 3 injections (43% of cases with a known injection number; Table 2), but did occur after a wide range of 1–94 injections.

**Table 2:** **PDSS cases in routine clinical practice according to number of injections and time to onset**

**Parameter**	**Percentage of cases**
**Number of injections** ^**a**^	
1–3	43 (83/193)
4–9	30 (58/193)
10–20	13 (26/193)
>20	13 (26/193)
Unknown	145/338
**Time to onset of PDSS** ^**b**^	
≤1 hour	91 (294/323)
1–2 hours	7 (24/323)
2–3 hours	1 (4/323)
Precise time unknown but within 3-hour observation period	1/338
Time unknown	15/338

The table shows the percentage of cases of PDSS according to the number of injections received at the time the syndrome occurred, and according to time to onset after injection, in two post-marketing safety studies and spontaneous reports.

^a^The number of injections received before PDSS occurred was known for 193 of the 338 cases.

^b^The precise time to onset of PDSS was known for 323 of the 338 cases.

PDSS = post-injection delirium/sedation syndrome.

### Time to initial onset of PDSS

**Figure 1:**
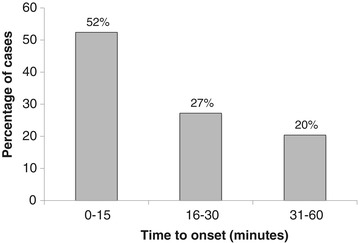
**Time to onset of PDSS among cases presenting in the first 60 minutes after injection.** The figure illustrates the percentage of cases of PDSS in routine clinical practice according to time to onset among 294 cases presenting in the first 60 minutes after injection. PDSS = post-injection delirium/sedation syndrome.

For both the post-marketing safety studies and spontaneous data, the longest reported time to onset of PDSS was 173 minutes. Since the launch of olanzapine LAI, no cases of PDSS have been reported beyond 180 minutes after injection. Approximately 91% of cases of PDSS presented within the first 60 minutes (Table 2). Of the 294 cases identified in the first 60 minutes, 52% (154/294) presented within the first 15 minutes (Figure 1). Time to onset of PDSS was unknown in 15 cases.

### Outcomes of PDSS events

For both the post-marketing safety studies and spontaneous data, there were no fatal outcomes. Injections were administered in healthcare facilities outside a hospital setting in 93% of cases (316/338 cases), and in hospital in 7% of cases (22/338). Of the 316 cases receiving an injection outside hospital, 65% (206/316) required subsequent hospitalisation as a result of PDSS events. Patients who developed PDSS following an in-hospital injection (22 cases) remained in hospital; it is not known whether these events in themselves would have merited hospitalisation or an extension of hospitalisation. Twelve percent (39/338) of the cases of PDSS were treated in an Emergency Department with no report of subsequent hospitalisation. Eighteen percent of cases (60/338) did not require hospitalisation. The duration of hospitalisation was known for 44% (149/338) of the hospitalised cases; in 52% (77/149) of these it was ≤24 hours.

Management of PDSS was documented for 72% (243/338) of cases; a large number of these cases required either no treatment or only continued monitoring with or without intravenous fluids (Table 3). Medications were administered as a treatment for PDSS in 31% (75/243) of cases, and included beta-agonists, benzodiazepines, diuretics and anticholinergics. A sedative drug (benzodiazepine or antipsychotic) was given in 19% (45/243) of cases. A small percentage of cases (5%; 13/243) required intubation/ventilation. This was administered prophylactically in approximately half of the cases (6 cases: 5 due to the patient being unconscious/comatose and 1 due to the patient requiring sedation for severe agitation) and for respiratory distress in the remaining cases (7 cases: 6 as a result of airway blockage or aspiration, and 1 as a result of poor oxygenation due to severe agitation). Data on continuation of olanzapine LAI was available in 62% (210/338) of cases, and the drug was continued in 55% of these cases (116/210).

**Table 3:** **Treatments administered during PDSS events in routine clinical practice**

**Treatment**	**Percentage of cases** ^**a**^
No treatment	19 (47/243)
Medication	31 (75/243)
Potentially sedative drug	19 (45/243)
Intravenous fluids	25 (60/243)
Monitoring	21 (51/243)
Oxygen	5 (13/243)
Physical restraint	4 (9/243)
Admission to intensive care unit	18 (44/243)
Ventilation/intubation	5 (13/243)

The table shows the treatment requirements for PDSS in the 243 cases where treatment data were available from two post-marketing safety studies and spontaneous reports.

^a^Some cases involved more than one type of treatment, and are therefore included more than once in the table.

PDSS = post-injection delirium/sedation syndrome.

Recovery status was reported for 94% (318/338) of PDSS cases; 98% (311/318) of patients with recovery status data had a full recovery which took ≤ 72 hours in 88% (273/311) of these cases. The time to recovery was unknown in 10% (30/311) of cases. Seven of the 318 patients with recovery status data were reported as not being recovered on the day of the report, and recovery outcome was unknown for the remaining 20 cases.

### Signs and symptoms of PDSS

**Figure 2:**
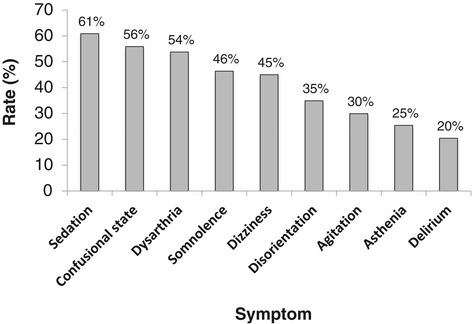
**Most frequently reported symptoms of PDSS in routine clinical practice.** The figure illustrates the most frequently reported symptoms, expressed as a percentage of the total number of PDSS events (n = 338), in routine clinical practice. PDSS = post-injection delirium/sedation syndrome.

In both the post-marketing safety studies and spontaneous reports, the symptoms of PDSS were consistent with those observed in clinical trials and those associated with an oral olanzapine overdose [4,5]. The most frequently observed symptoms were assessed as being related to delirium and excessive sedation (Figure 2). Delirium-related symptoms included ataxia, cognitive disorder, confusional state, delirium, disorientation, disturbance in attention, dysarthria and speech disorder. Delirium-related symptoms were reported in 86% (290/338) of cases, sedation-related symptoms in 89% (299/338) of cases and both types of symptoms in 74% (251/338) of cases. All cases of PDSS involved either sedation- or delirium-related symptoms.

Other clinical events reported in ≥5% of cases (expressed as a percentage of the 338 PDSS events) were dizziness (45%), agitation (30%), asthenia (25%), anxiety (18%), gait disturbance (17%), fatigue (17%), tachycardia (13%), restlessness (12%), hypertension (10%), akathisia (9%), aggression (9%), hypotension (8%), extrapyramidal symptoms (8%), increased heart rate (7%), malaise (7%), and nausea (6%).

## Discussion

The results of the current analysis show that the occurrence rate and clinical characteristics of PDSS in real-world clinical practice are consistent with those observed in formal clinical trials of olanzapine LAI, as summarised in Table 4. Indeed, the rate of PDSS per-injection appears to have remained consistent throughout the clinical trials and post-marketing use, at 0.05–0.09% of injections. The per-patient rate in clinical practice (0.46–1.03% of patients) and in clinical trials (1.4–2% of patients, based on reporting rates from spontaneous reports and incidence rates from post-marketing safety studies, respectively) were also generally consistent, taking into consideration the shorter length of commercial use at present relative to the longer average exposures in clinical trials, in which patients were treated for up to 6 years [5-8]. As a PDSS event can occur with any injection, the per-patient rate would be expected to increase as individual patients receive more injections.

**Table 4:** **Comparison of characteristics of PDSS in routine clinical practice and formal clinical trials** [5-8]

**Characteristic**	**Routine clinical practice**	**Formal clinical trials**
Rate of PDSS (% of injections)	0.07%	0.07–0.09%
Rate of PDSS (% of patients)	0.46–1.03%	1.4–2.0%
Cases starting within 60 minutes of injection	91%	80%
Cases requiring hospitalisation	65%	77%
Cases requiring medication/specific treatment	31%	37%
Cases showing recovery within 72 hours	88%	100%
Cases involving delirium-related AEs	86%	97%
Cases involving sedation-related AEs	89%	87%
Cases involving delirium & sedation-related AEs	74%	83%

AE = adverse event; PDSS = post-injection delirium/sedation syndrome.

In our analysis, the majority (91%) of PDSS cases presented within 60 minutes of injection and, of these, around half (52%) presented within the first 15 minutes. This is consistent with the timings observed in clinical trials, where 80% of cases occurred within 60 minutes of an injection, and the mean and median times to the onset of symptoms were 49 and 25 minutes, respectively [6]. In addition, the majority of cases of PDSS in our analysis (73%) were reported following the first 9 injections, and 43% of cases were reported following the first 3 injections. In clinical trials, the median injection number at which a PDSS event occurred was 21 [6].

The symptoms of PDSS in clinical practice were similar to those observed in previous clinical trials [6], with a clinical presentation consistent with symptoms of olanzapine overdose. As in the clinical trials [6], all cases in the present analysis included either sedation- or delirium-related symptoms, with most cases including both (Table 4). In the cases of PDSS reported by Detke et al. [6], no clinically significant decreases in blood pressure, heart rate or respiration were reported. In the present analyses, however, a small number of patients required intubation due to respiratory distress. In the post-marketing safety studies, a range of changes in blood pressure was noted, with some patients experiencing hypertension and others experiencing hypotension. However, changes in blood pressure are not considered a defining feature of PDSS [6]. It should also be noted that in 40% of cases in the clinical trials, the initial symptoms of PDSS were not related to sedation or delirium but presented as general malaise, anxiety, agitation or irritability. Thus, as the early symptoms of PDSS may not be immediately obvious and cannot be measured through changes in vital signs, continued patient observation offers the best approach for early detection of PDSS.

The outcomes for patients with PDSS in routine clinical practice were also similar to those in clinical trials: none of the PDSS events were fatal, and most resolved within 72 hours. Similarly, the signs and symptoms of PDSS in routine clinical practice were consistent with those observed in clinical trials, and with those reported in cases of olanzapine overdose [5,6]. It should be noted that two deaths were reported in patients taking olanzapine LAI, in whom post-mortem blood samples revealed elevated olanzapine concentrations [14]. However, neither case was adjudicated as being a PDSS event, as there was no evidence to support this possibility. Because post-mortem olanzapine concentrations in the blood tend to rise as a result of post-mortem redistribution from other sites in the body [15,16], the post-mortem data could not be considered to reflect the likely concentrations before death [17]. Nevertheless, it is important to be aware that the potential for death, although small, always exists with olanzapine overdose, and thus patients with PDSS should be managed with appropriate care.

As a result of the emergence of PDSS, prescribing information for olanzapine LAI contains appropriate warnings and country-specific monitoring requirements. In addition, all prescribers of olanzapine LAI are required to undertake country-specific education and training to enable them to identify and manage the signs and symptoms of PDSS. Olanzapine LAI must be administered in a healthcare facility with ready access to emergency response services, and all patients are required to undergo observation for a period of time by an appropriately qualified healthcare professional after receiving an injection [7,8]. If PDSS is suspected, close medical supervision and monitoring should continue until examination indicates that the signs and symptoms have resolved. Since no clear risk factors and no concomitant medications have been identified as predictors of PDSS, observation must be undertaken in all patients after every injection [6-9].

As PDSS most likely occurs as a result of a portion of the olanzapine dose entering the bloodstream [9], use of the correct injection technique is important [6]. Consequently, prescribers of olanzapine LAI are also required to receive training on the correct injection technique. Accidental intravascular injection is a known risk for all drugs that are administered by intramuscular injection, and has been reported with penicillins, promethazine, benzodiazepines and barbiturates [10-13].

Limitations of the current analysis include the likelihood of under-reporting for spontaneous data, which rely on patients and/or healthcare providers contacting Eli Lilly or regulatory authorities. In addition, the identification of PDSS from spontaneous reports relies on sufficient details being provided by the person reporting the case [18]. Thus, while some cases reported as possible PDSS may not have been adjudicated as being a confirmed PDSS case because the reported symptoms did not meet the established criteria, others could not be confirmed because of insufficient information. Also, because exact denominators (total number of patients and total number of injections) are not known, rate calculations rely on the use of estimates. Thus, the results from spontaneous data should be viewed with caution. By contrast, data from the formal post-marketing safety studies are more accurate and of higher quality as they are collected from a known number of patients receiving a known number of injections using a standard reporting format. Nevertheless, despite the limitations of spontaneous reporting data, the per-injection rates of PDSS are almost identical across the various sources, supporting the probable validity of the spontaneous reporting findings.

Another limitation is that longer exposures occurred in the clinical trials than are yet available during post-marketing use; however, substantially greater numbers of patients have now been exposed in post-marketing use (over 60,000) than in the clinical trials (2,054 [6]).

Despite these limitations, the rate, clinical presentation and outcomes of PDSS were generally consistent with those seen in clinical trials. This suggests that there is good awareness of PDSS among healthcare providers and that the PDSS educational programme for healthcare providers is encouraging active identification and management of the syndrome.

## Conclusions

Olanzapine LAI has been in routine clinical use for over 5 years, and a large body of safety data is now available from a number of different countries and a variety of clinical settings. The current analysis has shown that the rate (both per-injection and per-patient), outcome and presentation of PDSS events being reported during routine clinical use of olanzapine LAI are similar to those reported in clinical trials. To date there have been no fatalities associated with confirmed PDSS events, and most events continue to appear temporary in nature, typically resolving in less than 72 hours. Adherence to the labelled conditions and precautions of use as well as risk-minimisation measures will continue to allow appropriate identification, diagnosis and management of PDSS cases.

## Endnote

^a^Other signs and symptoms not listed under 1a may occur with olanzapine overdose but are not considered criteria for PDSS. See olanzapine prescribing information.

## Additional file

Additional file 1:
**Ethical Review Boards for Trial F1D-MC-B034.**
