# Supplementary material for: Olanzapine long-acting injection: a review of first experiences of post-injection delirium/sedation syndrome in routine clinical practice
Source: BMC Psychiatry. 2015 Apr 2;15:65. doi: 10.1186/s12888-015-0450-9 (PMC4389990; doi:10.1186/s12888-015-0450-9)
Supplement: Additional file 1: — Ethical Review Boards for Trial F1D-MC-B034. [file 12888_2015_450_MOESM1_ESM.docx]

**Ethical Review Boards for Trial F1D-MC-B034**

| **Trial Country Name** | **ERB Name, Address, City, State/Country/Province, Postal Code, Country** |
| --- | --- |
| AUSTRIA | Ethikkommission Med. Univ. Und Akh Wien Borschkegasse 8b/6 Wien, Wien, A-1090, Austria |
| AUSTRIA | Ethikkommission Med. Univ. Und Akh Wien Borschkegasse 8b/6 Wien, Wien, A-1090, Austria |
| AUSTRIA | Ethikkommission Med. Univ. Und Akh Wien Borschkegasse 8b/6 Wien, Wien, A-1090, Austria |
| BELGIUM | Centre Hospitalier Regional De La Citadelle Boulevard De 12e De Ligne 1 Liege, 4000, Belgium |
| BELGIUM | Centre Hospitalier Regional De La Citadelle Boulevard De 12e De Ligne 1 Liege, 4000, Belgium |
| BELGIUM | Centre Hospitalier Regional De La Citadelle Boulevard De 12e De Ligne 1 Liege, 4000, Belgium |
| BELGIUM | Centre Hospitalier Regional De La Citadelle Boulevard De 12e De Ligne 1 Liege, 4000, Belgium |
| BELGIUM | Centre Hospitalier Regional De La Citadelle Boulevard De 12e De Ligne 1 Liege, 4000, Belgium |
| BELGIUM | Centre Hospitalier Regional De La Citadelle Boulevard De 12e De Ligne 1 Liege, 4000, Belgium |
| BELGIUM | Centre Hospitalier Regional De La Citadelle Boulevard De 12e De Ligne 1 Liege, 4000, Belgium |
| BULGARIA | Ethics Committee For Multicentre Trials 5, Sveta Nedelya Square Sofia, Balkana, 1000, Bulgaria |
| BULGARIA | Ethics Committee For Multicentre Trials 5, Sveta Nedelya Square Sofia, Balkana, 1000, Bulgaria |
| BULGARIA | Ethics Committee For Multicentre Trials 5, Sveta Nedelya Square Sofia, Balkana, 1000, Bulgaria |
| BULGARIA | Ethics Committee For Multicentre Trials 5, Sveta Nedelya Square Sofia, Balkana, 1000, Bulgaria |
| BULGARIA | Ethics Committee For Multicentre Trials 5, Sveta Nedelya Square Sofia, Balkana, 1000, Bulgaria |
| BULGARIA | Ethics Committee For Multicentre Trials 5, Sveta Nedelya Square Sofia, Balkana, 1000, Bulgaria |
| CROATIA | Sredisnje Eticko Povjerenstvo Ksaverska Cesta 4 Zagreb, 10000, Croatia |
| CROATIA | Sredisnje Eticko Povjerenstvo Ksaverska Cesta 4 Zagreb, 10000, Croatia |
| FINLAND | Research Ethics Committee Of Helsinki University Hospital Biomedicum Helsinki 2 CP.o.box 705 Helsinki, 00290 HUS, Finland |
| GERMANY | Ethikkommission Der Landesärztekammer HessenIm Vogelsgesang 3 Frankfurt Am Main, Hessen, 60488, Germany |
| GERMANY | Ethikkommission Der Landesärztekammer HessenIm Vogelsgesang 3 Frankfurt Am Main, Hessen, 60488, Germany |
| GERMANY | Ethikkommission Der Landesärztekammer HessenIm Vogelsgesang 3 Frankfurt Am Main, Hessen, 60488, Germany |
| GERMANY | Ethikkommission Der Landesärztekammer HessenIm Vogelsgesang 3 Frankfurt Am Main, Hessen, 60488, Germany |
| GERMANY | Ethikkommission Der Landesärztekammer HessenIm Vogelsgesang 3 Frankfurt Am Main, Hessen, 60488, Germany |
| GERMANY | Ethikkommission Der Landesärztekammer HessenIm Vogelsgesang 3 Frankfurt Am Main, Hessen, 60488, Germany |
| GERMANY | Ethikkommission Der Landesärztekammer HessenIm Vogelsgesang 3 Frankfurt Am Main, Hessen, 60488, Germany |
| GERMANY | Ethikkommission Der Landesärztekammer HessenIm Vogelsgesang 3 Frankfurt Am Main, Hessen, 60488, Germany |
| GERMANY | Ethikkommission Der Landesärztekammer HessenIm Vogelsgesang 3 Frankfurt Am Main, Hessen, 60488, Germany |
| GERMANY | Ethikkommission Der Landesärztekammer HessenIm Vogelsgesang 3 Frankfurt Am Main, Hessen, 60488, Germany |
| GERMANY | Ethikkommission Der Landesärztekammer HessenIm Vogelsgesang 3 Frankfurt Am Main, Hessen, 60488, Germany |
| GERMANY | Ethikkommission Der Landesärztekammer HessenIm Vogelsgesang 3 Frankfurt Am Main, Hessen, 60488, Germany |
| GERMANY | Ethikkommission Der Landesärztekammer HessenIm Vogelsgesang 3 Frankfurt Am Main, Hessen, 60488, Germany |
| GERMANY | Ethikkommission Der Landesärztekammer HessenIm Vogelsgesang 3 Frankfurt Am Main, Hessen, 60488, Germany |
| GERMANY | Ethikkommission Der Landesärztekammer HessenIm Vogelsgesang 3 Frankfurt Am Main, Hessen, 60488, Germany |
| GERMANY | Ethikkommission Der Landesärztekammer HessenIm Vogelsgesang 3 Frankfurt Am Main, Hessen, 60488, Germany |
| GERMANY | Ethikkommission Der Landesärztekammer HessenIm Vogelsgesang 3 Frankfurt Am Main, Hessen, 60488, Germany |
| GERMANY | Ethikkommission Der Landesärztekammer HessenIm Vogelsgesang 3 Frankfurt Am Main, Hessen, 60488, Germany |
| GERMANY | Ethikkommission Der Landesärztekammer HessenIm Vogelsgesang 3 Frankfurt Am Main, Hessen, 60488, Germany |
| GERMANY | Ethikkommission Der Landesärztekammer HessenIm Vogelsgesang 3 Frankfurt Am Main, Hessen, 60488, Germany |
| GERMANY | Ethikkommission Der Landesärztekammer HessenIm Vogelsgesang 3 Frankfurt Am Main, Hessen, 60488, Germany |
| GERMANY | Ethikkommission Der Landesärztekammer HessenIm Vogelsgesang 3 Frankfurt Am Main, Hessen, 60488, Germany |
| GERMANY | Ethikkommission Der Landesärztekammer HessenIm Vogelsgesang 3 Frankfurt Am Main, Hessen, 60488, Germany |
| GERMANY | Ethikkommission Der Landesärztekammer HessenIm Vogelsgesang 3 Frankfurt Am Main, Hessen, 60488, Germany |
| GERMANY | Ethikkommission Der Landesärztekammer HessenIm Vogelsgesang 3 Frankfurt Am Main, Hessen, 60488, Germany |
| GERMANY | Ethikkommission Der Landesärztekammer HessenIm Vogelsgesang 3 Frankfurt Am Main, Hessen, 60488, Germany |
| GERMANY | Ethikkommission Der Landesärztekammer HessenIm Vogelsgesang 3 Frankfurt Am Main, Hessen, 60488, Germany |
| GERMANY | Ethikkommission Der Landesärztekammer HessenIm Vogelsgesang 3 Frankfurt Am Main, Hessen, 60488, Germany |
| GERMANY | Ethikkommission Der Landesärztekammer HessenIm Vogelsgesang 3 Frankfurt Am Main, Hessen, 60488, Germany |
| GERMANY | Ethikkommission Der Landesärztekammer HessenIm Vogelsgesang 3 Frankfurt Am Main, Hessen, 60488, Germany |
| GERMANY | Ethikkommission Der Landesärztekammer HessenIm Vogelsgesang 3 Frankfurt Am Main, Hessen, 60488, Germany |
| GERMANY | Ethikkommission Der Landesärztekammer HessenIm Vogelsgesang 3 Frankfurt Am Main, Hessen, 60488, Germany |
| GERMANY | Ethikkommission Der Landesärztekammer HessenIm Vogelsgesang 3 Frankfurt Am Main, Hessen, 60488, Germany |
| GERMANY | Ethikkommission Der Landesärztekammer HessenIm Vogelsgesang 3 Frankfurt Am Main, Hessen, 60488, Germany |
| GERMANY | Ethikkommission Der Landesärztekammer HessenIm Vogelsgesang 3 Frankfurt Am Main, Hessen, 60488, Germany |
| GERMANY | Ethikkommission Der Landesärztekammer HessenIm Vogelsgesang 3 Frankfurt Am Main, Hessen, 60488, Germany |
| GERMANY | Ethikkommission Der Landesärztekammer HessenIm Vogelsgesang 3 Frankfurt Am Main, Hessen, 60488, Germany |
| GERMANY | Ethikkommission Der Landesärztekammer HessenIm Vogelsgesang 3 Frankfurt Am Main, Hessen, 60488, Germany |
| GERMANY | Ethikkommission Der Landesärztekammer HessenIm Vogelsgesang 3 Frankfurt Am Main, Hessen, 60488, Germany |
| GREECE | General Hospital Of Katerini 6th Klm Katerinis – Arona Katerini, Pieria, 60100, Greece |
| GREECE | Mental Hospital Of Thessaloniki Lagada 196,Thessaloniki, Stavroupolis, 56429, Greece |
| GREECE | Mental Hospital Of Thessaloniki Lagada 196, Stavroupolis Thessaloniki, Thessaloniki, 546 30, Greece |
| GREECE | General Hospital Of Volos Achillopouleio Polimeri 134 Volos, Magnisia, 38222, Greece |
| GREECE | Venizeleio-pananeio General Hospital Of Heraklion Leoforos Knossou Scientific Committee Iraklion, 71000, Greece |
| GREECE | Attikon' University Hospital 1, Rimini Str.Haidari/Athens, 12462, Greece |
| GREECE | Papageorgiou Regional General Hospital Of Thessaloniki Ring Road Of Thessaloniki - N.efkarpia Thessaloniki, 56429, Greece |
| GREECE | Mental Hospital Of Thessaloniki Lagada 196, Thessaloniki, Stavroupolis, 56429, Greece |
| GREECE | Mental Hospital Of Athens "dafni" 374, Athinon Ave Chaidari, Attiki, 12462, Greece |
| GREECE | Panarkadiko General Hospital Of Tripolis Erithrou Stavrou Terma Tripolis, Arkadia, 22100, Greece |
| GREECE | Mental Hospital Of Athens "dafni" 374, Athinon Ave Chaidari, Attiki, 12462, Greece |
| GREECE | Mental Hospital Of Thessaloniki Lagada 196, Stavroupolis Thessaloniki, Thessaloniki, 546 30, Greece |
| GREECE | Sotiria General Hospital 152 Mesogion Avenue Athens, 11527, Greece |
| GREECE | Mental Hospital Of Kerkyra Plateia Tsirigoti Corfu, 49100, Greece |
| GREECE | University Hospital Of Ioannina Stavros Niarchos Avenue Ioannina, Ioannina, 45500, Greece |
| GREECE | General Hospital Of Arta Lofos Peranthis Arta, Arta, 47100, Greece |
| GREECE | Mental Hospital Of Thessaloniki Lagada 196, Thessaloniki, Stavroupolis, 56429, Greece |
| GREECE | Ahepa Hospital St. Kyriakidi 1 Thessaloniki, 54636, Greece |
| GREECE | Eginition Hospital Of Athens 72 Vas. Sophia's Avenue Athens, 11528, Greece |
| GREECE | Mental Hospital Of Thessaloniki Lagada 196, Stavroupolis Thessaloniki, Thessaloniki, 54630, Greece |
| GREECE | General Hospital Of Agrinio Afon Kentrou 7 Agrinio, Agrinio, 30100, Greece |
| GREECE | General Hospital Of Patras "agios Andreas" Tsertidou 1 Patra, Achaia, 26335, Greece |
| GREECE | General Hospital Of Nikaias Piraeus - Agios Panteleimon Fanarioton 3 - Aspra Homata Scientific Committee Nikaia-piraeus, 18454, Greece |
| GREECE | General Hospital Of Nikaias Piraeus - Agios Panteleimon Fanarioton 3 - Aspra Homata Scientific Committee Nikaia-piraeus, 18454, Greece |
| GREECE | Mental Hospital Of Athens "dafni" 374, Athinon Ave Chaidari, Attiki, 12462, Greece |
| GREECE | Mental Hospital Of Athens "dafni" Leoforos Athinon (kavalas) 360 Haidari, Athens, 12462, Greece |
| GREECE | Chania General Hospital 'agios Georgios' Mournies Chania, Greece, 73300, Greece |
| GREECE | University General Hospital Of Heraklion Stavrakia And Voutes.Heraklion, Crete, 71110, Greece |
| HUNGARY | Egeszsegugyi Tudomanyos Tanacs Arany Janos U. 6-8 Budapest,1051, Hungary |
| HUNGARY | Egeszsegugyi Tudomanyos Tanacs Arany Janos U. 6-8 Budapest,1051, Hungary |
| HUNGARY | Egeszsegugyi Tudomanyos Tanacs Arany Janos U. 6-8 Budapest, 1051, Hungary |
| HUNGARY | Egeszsegugyi Tudomanyos Tanacs Arany Janos U. 6-8 Budapest, 1051, Hungary |
| HUNGARY | Egeszsegugyi Tudomanyos Tanacs Arany Janos U. 6-8 Budapest, 1051, Hungary |
| HUNGARY | Egeszsegugyi Tudomanyos Tanacs Arany Janos U. 6-8 Budapest, 1051, Hungary |
| HUNGARY | Egeszsegugyi Tudomanyos Tanacs Arany Janos U. 6-8 Budapest, 1051, Hungary |
| HUNGARY | Egeszsegugyi Tudomanyos Tanacs Arany Janos U. 6-8 Budapest, 1051, Hungary |
| HUNGARY | Egeszsegugyi Tudomanyos Tanacs Arany Janos U. 6-8 Budapest, 1051, Hungary |
| HUNGARY | Egeszsegugyi Tudomanyos Tanacs Arany Janos U. 6-8 Budapest, 1051, Hungary |
| HUNGARY | Egeszsegugyi Tudomanyos Tanacs Arany Janos U. 6-8 Budapest, 1051, Hungary |
| HUNGARY | Egeszsegugyi Tudomanyos Tanacs Arany Janos U. 6-8 Budapest, 1051, Hungary |
| HUNGARY | Egeszsegugyi Tudomanyos Tanacs Arany Janos U. 6-8 Budapest, 1051, Hungary |
| HUNGARY | Egeszsegugyi Tudomanyos Tanacs Arany Janos U. 6-8 Budapest, 1051, Hungary |
| HUNGARY | Egeszsegugyi Tudomanyos Tanacs Arany Janos U. 6-8 Budapest, 1051, Hungary |
| HUNGARY | Egeszsegugyi Tudomanyos Tanacs Arany Janos U. 6-8 Budapest, 1051, Hungary |
| HUNGARY | Egeszsegugyi Tudomanyos Tanacs Arany Janos U. 6-8 Budapest, 1051, Hungary |
| HUNGARY | Egeszsegugyi Tudomanyos Tanacs Arany Janos U. 6-8 Budapest, 1051, Hungary |
| HUNGARY | Egeszsegugyi Tudomanyos Tanacs Arany Janos U. 6-8 Budapest, 1051, Hungary |
| HUNGARY | Egeszsegugyi Tudomanyos Tanacs Arany Janos U. 6-8 Budapest, 1051, Hungary |
| HUNGARY | Egeszsegugyi Tudomanyos Tanacs Arany Janos U. 6-8 Budapest, 1051, Hungary |
| HUNGARY | Egeszsegugyi Tudomanyos Tanacs Arany Janos U. 6-8 Budapest, 1051, Hungary |
| HUNGARY | Egeszsegugyi Tudomanyos Tanacs Arany Janos U. 6-8 Budapest, 1051, Hungary |
| HUNGARY | Egeszsegugyi Tudomanyos Tanacs Arany Janos U. 6-8 Budapest, 1051, Hungary |
| HUNGARY | Egeszsegugyi Tudomanyos Tanacs Arany Janos U. 6-8Budapest, 1051, Hungary |
| HUNGARY | Egeszsegugyi Tudomanyos TanacsArany Janos U. 6-8 Budapest, 1051, Hungary |
| IRELAND | London - South East Charing Cross Hospital Fulham Palace Road, London, W6 8RF, United Kingdom |
| IRELAND | London - South East Charing Cross Hospital Fulham Palace Road, London, W6 8RF, United Kingdom |
| ITALY | Comitato Etico Dell`azienda Osped-univ Riuniti Di Foggia Viale Luigi Pinto Foggia, 71100, Italy |
| ITALY | Comitato Etico Della Asl Di Caserta Via Unita Italiana N. 28 Caserta, Caserta, 81100, Italy |
| ITALY | Comitato Etico Azienda Ospedaliera San Gerardo Via Pergolesi N. 33 Monza, Monza, 20052, Italy |
| ITALY | Comitato Etico Campania Sud Presso Asl Napoli 3 Sud Piazza San Giovanni, 7 Brusciano, Napoli, 80031, Italy |
| ITALY | Comitato Etico Lazio Ii Presso Azienda Usl Roma CViale Dell 'arte, 68 Roma, Roma, 00144, Italy |
| ITALY | Comitato Etico Dell'azienda Ospedaliera Di Cosenza Via San Martino Cosenza, Cosenza, 87100, Italy |
| ITALY | Ce Dell' Azienda Policlinico Umberto L Di Roma Viale Del Policlinico, 155 Roma, 00161, Italy |
| ITALY | Comitato Etico Milano Area B Irccs Policlinico Via Francesco Sforza 28 Milano, 20122, Italy |
| ITALY | Comitato Etico Dell ' Azienda Usl Br/1 Di BrindisiVia Napoli, 8 Brindisi, 72100, Italy |
| ITALY | Comitato Etico Lazio L Presso Ao S.camillo-forlanini Circonvallazione Gianicolense, 87 Roma, Roma, 00152, Italy |
| ITALY | Comitato Etico Ao 'citta Della Salute E Della Scienza Corso Bramante,88 Torino, Torino, 10126, Italy |
| SLOVAKIA | Eticka Komisia Bratislavskeho Samospravneho Kraja Sabinovska 16 Po Box 106 Bratislava, Slovak Republic, 820 05, Slovakia |
| SLOVENIA | Komisija Rs Za Medicinsko Etiko Zaloska 7 Ljubljana, 1525, Slovenia |
| SLOVENIA | Komisija Rs Za Medicinsko Etiko Zaloska 7 Ljubljana, 1525, Slovenia |
| SLOVENIA | Komisija Rs Za Medicinsko Etiko Zaloska 7 Ljubljana, 1525, Slovenia |
| SPAIN | Hospital Sant PauAvda. Sant Antoni Maria Claret, 167 Barcelona, Cataluna, 08025, Spain |
| SWEDEN | Regionala Etikproevningsnaemnden I Stockholm Fe 289 Karolinska Institutet, Stockholm, 17177, Sweden |
| SWEDEN | Regionala Etikproevningsnaemnden I Stockholm Fe 289 Karolinska Institutet, Stockholm, 17177, Sweden |
| SWEDEN | Regionala Etikproevningsnaemnden I Stockholm Fe 289 Karolinska Institutet, Stockholm, 17177, Sweden |
| SWEDEN | Regionala Etikproevningsnaemnden I Stockholm Fe 289 Karolinska Institutet, Stockholm, 17177, Sweden |
| SWEDEN | Regionala Etikproevningsnaemnden I Stockholm Fe 289 Karolinska Institutet, Stockholm, 17177, Sweden |
| SWEDEN | Regionala Etikproevningsnaemnden I Stockholm Fe 289 Karolinska Institutet, Stockholm, 17177, Sweden |
| SWEDEN | Regionala Etikproevningsnaemnden I Stockholm Fe 289 Karolinska Institutet, Stockholm, 17177, Sweden |
| SWEDEN | Regionala Etikproevningsnaemnden I Stockholm Fe 289 Karolinska Institutet, Stockholm, 17177, Sweden |
| UNITED KINGDOM | London - South East Charing Cross Hospital, Fulham Palace Road, London, W6 8RF, United Kingdom |
